# Supplementary material for: Skilful forecasting of global fire activity using seasonal climate predictions
Source: Nat Commun. 2018 Jul 13;9:2718. doi: 10.1038/s41467-018-05250-0 (PMC6045620; doi:10.1038/s41467-018-05250-0)
Supplement: Supplementary file 1 — Supplementary Information [file 41467_2018_5250_MOESM1_ESM.pdf]

SUPPLEMENTARY INFORMATION
Skilful forecasting of global fire activity using seasonal climate predictions

Marco Turco[1,*], Sonia Jerez[2], Francisco J. Doblas-Reyes[3,4], Amir AghaKouchak[5], Maria Carmen Llasat[1], and Antonello Provenzale[6]

[1]University of Barcelona, Department of Applied Physics, 08028 Barcelona, Spain
[2]Regional Atmospheric Modeling Group, Department of Physics, University of Murcia, 30100 Murcia, Spain
[3]Barcelona Supercomputing Center (BSC), 08034 Barcelona, Spain
[4]ICREA, 08010 Barcelona, Spain
[5]Center for Hydrometeorology and Remote Sensing, Department of Civil and Environmental Engineering, University of California, Irvine, CA, 92697, USA
[6]Institute of Geosciences and Earth Resources (IGG), National Research Council (CNR), 56124 Pisa, Italy
[*]e.mail: turco.mrc@gmail.com

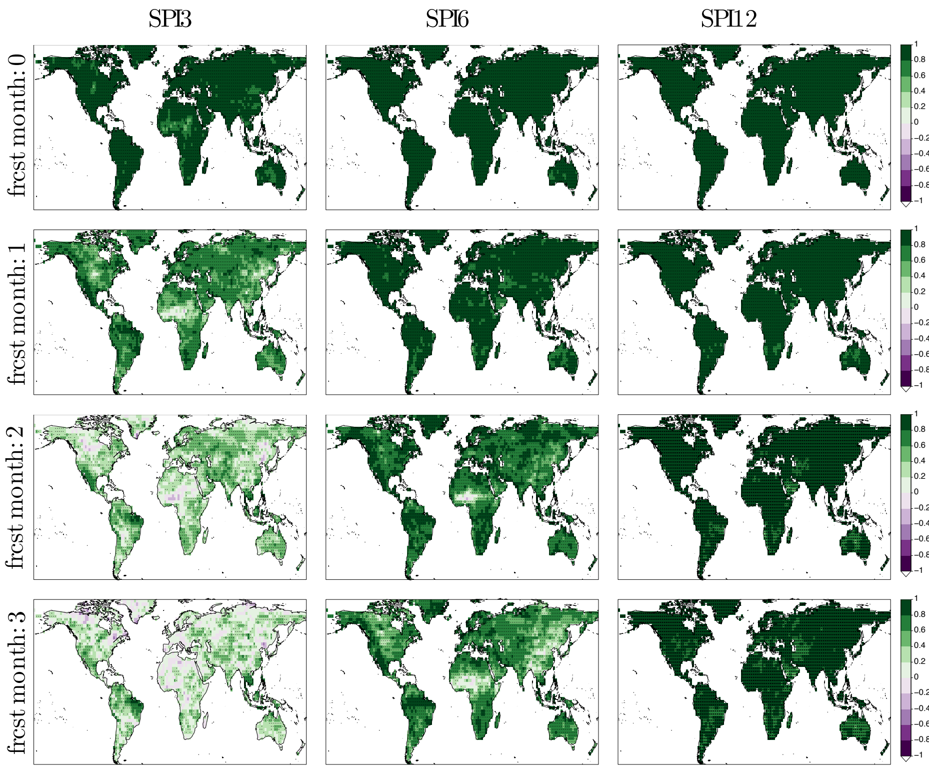

Supplementary Figure 1: Correlation of BESTENS forecasts wrt observed SPI for different forecast months (from 0 to +3) and different accumulation time scales (3, 6 and 12) over the period 1981-2016. The forecasts are initialized in February. Black circles indicate correlations that are significant (pvalue < 0.05).

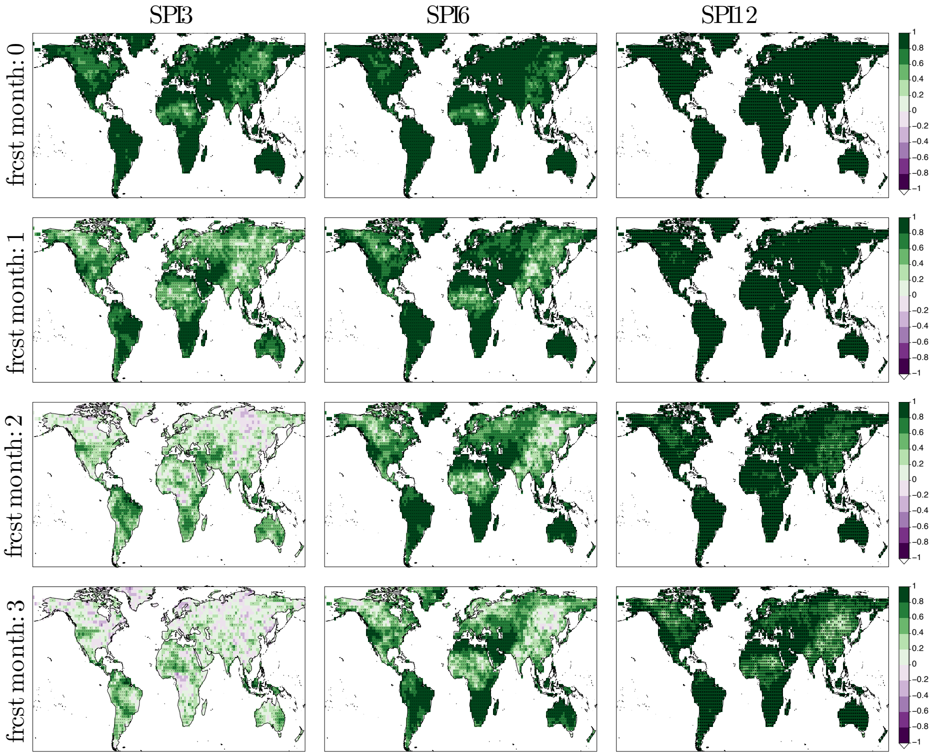

Supplementary Figure 2: Correlation of BESTENS forecasts wrt observed SPI for different forecast months (from 0 to +3) and different accumulation time scales (3, 6 and 12) over the period 1981-2015. The forecasts are initialized in May. Black circles indicate correlations that are significant (pvalue < 0.05).

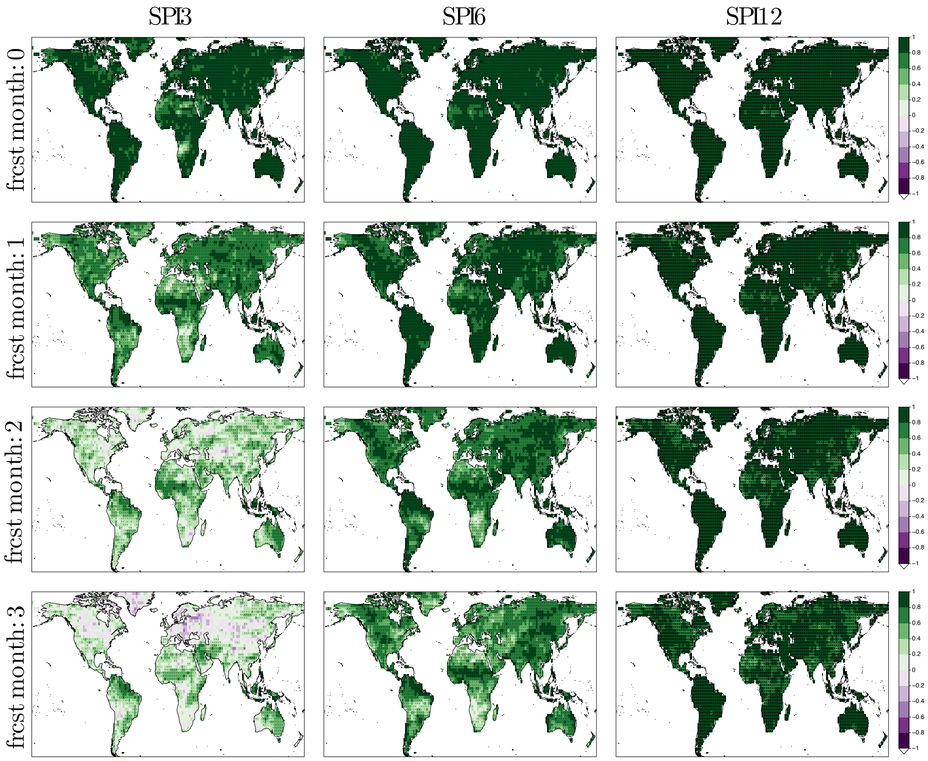

Supplementary Figure 3: Correlation of BESTENS forecasts wrt observed SPI for different forecast months (from 0 to +3) and different accumulation time scales (3, 6 and 12) over the period 1981-2015. The forecasts are initialized in August. Black circles indicate correlations that are significant (pvalue < 0.05).

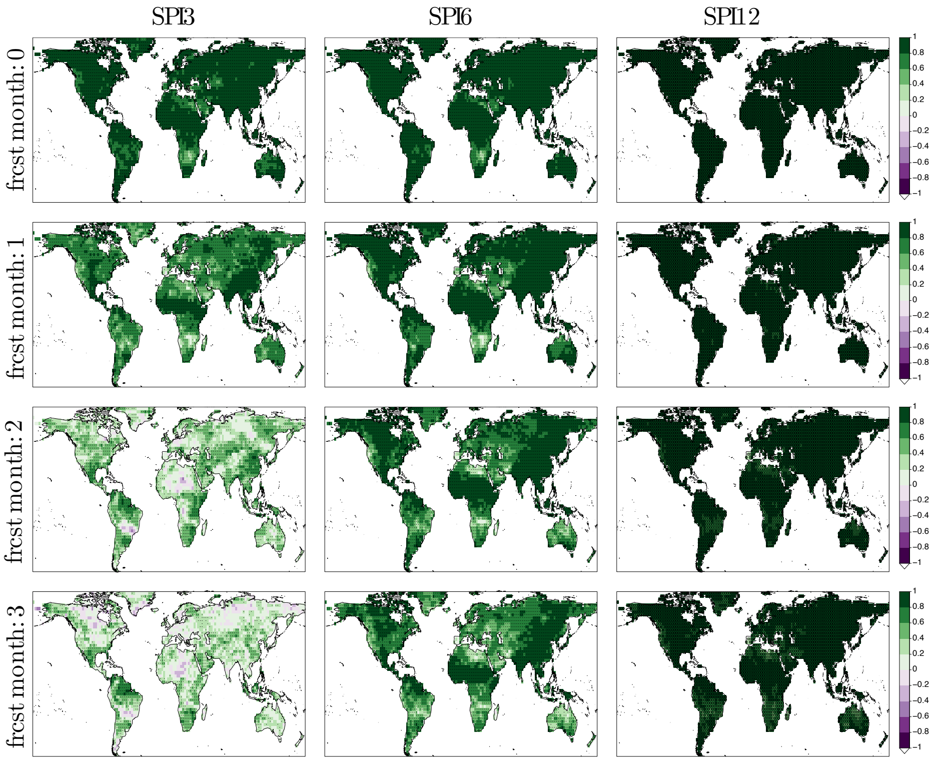

Supplementary Figure 4: Correlation of BESTENS forecasts wrt observed SPI for different forecast months (from 0 to +3) and different accumulation time scales (3, 6 and 12) over the period 1981-2016. The forecasts are initialized in November. Black circles indicate correlations that are significant (pvalue < 0.05).

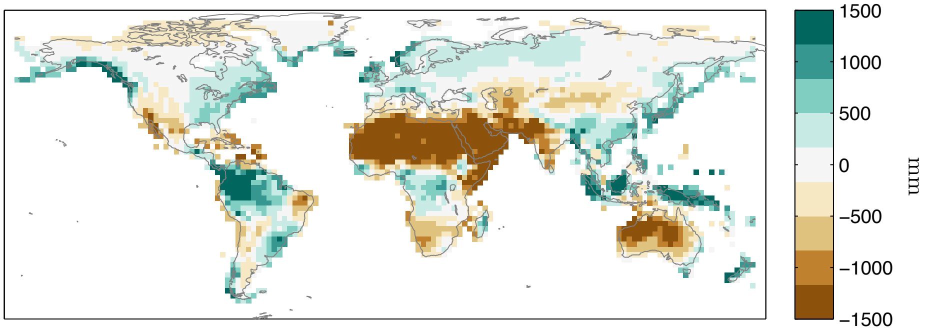

Supplementary Figure 5: Spatial distribution of the average annual water balance (precipitation minus potential evapotranspiration) for the period June 1995 - May 2016.

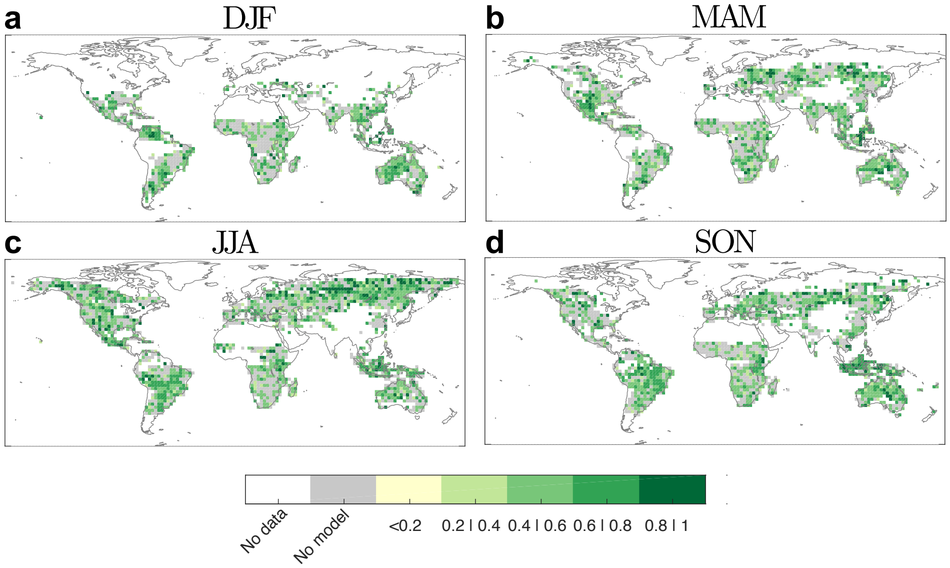

Supplementary Figure 6: Skill of BA predictions obtained using observed climate without detrending the data. Correlations of out-of-sample BA predictions using the SPI-BA model with observed data for (a) December-January-February (DJF), (b) March-April-May, (c) June-July-August (JJA) and (d) September-October-November (SON). Only correlations that are significant (p-value < 0.05) are shown.
